# Quantitative Three-Dimensional Assessment of the Pharmacokinetic Parameters of Intra- and Peri-tumoural Tissues on Breast Dynamic Contrast-Enhanced Magnetic Resonance Imaging

**DOI:** 10.1007/s10278-021-00509-3

**Published:** 2021-09-10

**Authors:** A. Niukkanen, H. Okuma, M. Sudah, P. Auvinen, A. Mannermaa, T. Liimatainen, R. Vanninen

**Affiliations:** 1 Department of Clinical Radiology, Diagnostic Imaging Center, Kuopio University Hospital, PO BOX 100, 70029 KYS, Kuopio, Finland; 2 Institute of Clinical Medicine, School of Medicine, Clinical Radiology, University of Eastern Finland, Kuopio, Finland; 3 Institute of Clinical Medicine, School of Medicine, Oncology, University of Eastern Finland, Kuopio, Finland; 4 Institute of Clinical Medicine, Pathology and Forensic Medicine, University of Eastern Finland, Kuopio, Finland; 5 Physics and Technology, Research Unit of Medical Imaging, University of Oulu, Oulu, Finland; 6 Department of Radiology, Oulu University Hospital, Oulu, Finland

**Keywords:** Dynamic contrast-enhanced, MRI, Breast cancer, Peri-tumoural, Reference tissue model, Pharmacokinetic

## Abstract

We aimed to assess the feasibility of three-dimensional (3D) segmentation and to investigate whether semi-quantitative dynamic contrast-enhanced magnetic resonance imaging (DCE-MRI) parameters are associated with traditional prognostic factors for breast cancer. In addition, we evaluated whether both intra-tumoural and peri-tumoural DCE parameters can differentiate the breast cancers that are more aggressive from those that are less aggressive. Consecutive patients with newly diagnosed invasive breast cancer and structural breast MRI (3.0 T) were included after informed consent. Fifty-six patients (mean age, 57 years) with mass lesions of > 7 mm in diameter were included. A semi-automatic image post-processing algorithm was developed to measure 3D pharmacokinetic information from the DCE-MRI images. The kinetic parameters were extracted from time-signal curves, and the absolute tissue contrast agent concentrations were calculated with a reference tissue model. Markedly, higher intra-tumoural and peri-tumoural tissue concentrations of contrast agent were found in high-grade tumours (*n* = 44) compared to low-grade tumours (*n* = 12) at every time point (*P* = 0.006–0.040), providing positive predictive values of 90.6–92.6% in the classification of high-grade tumours. The intra-tumoural and peri-tumoural signal enhancement ratios correlated with tumour grade, size, and Ki67 activity. The intra-observer reproducibility was excellent. We developed a model to measure the 3D intensity data of breast cancers. Low- and high-grade tumours differed in their intra-tumoural and peri-tumoural enhancement characteristics. We anticipate that pharmacokinetic parameters will be increasingly used as imaging biomarkers to model and predict tumour behavior, prognoses, and responses to treatment.

## Introduction

Dynamic contrast-enhanced magnetic resonance imaging (DCE-MRI) has been used extensively in oncological imaging for decades. DCE-MRI allows for malignant and benign tumours in the breast to be distinguished based on differences in the contrast agent enhancement patterns, and thus, the method improves diagnostic accuracy, with proven importance in differential diagnostics and pre-operative evaluation [1]. To evaluate the enhancement characteristics of lesions over time, a time intensity curve (TIC) is calculated by defining the region of interest (ROI) on the most suspicious region of enhancement within a lesion as instructed by the American College of Radiology Breast Imaging Reporting and Data System (ACR BI-RADS) [2]. The initial phase of the TIC is divided into slow, medium, and fast enhancement, and the delayed phase is divided into the persistent, plateau, and washout curves.

In DCE-MRI, the distribution of gadolinium contrast agent is compared between the vasculature and the intracellular and extracellular spaces at different time points. Therefore, the vascular density and the permeability of the vasculature in these tissues can be assessed, and the shape of the TIC can be determined. High permeability is linked to vascular leaking, which is attributed to tumour-growth-related angiogenesis [3]. Therefore, DCE-MRI-based parameters may be associated with the histopathological properties of tumours and may indicate their relative aggressiveness, and DCE-MRI may allow for higher precision pathophysiological assessment of tumours and the monitoring of therapeutic interventions [4].

Early breast cancer DCE-MRI studies detected an association between the TIC type, rapid initial enhancement, and microvessel density [5]. The link between imaging and histopathological characteristics was strong enough to sub-classify malignant breast tumours based on DCE-MRI [6]. Since these early discoveries, many new tools have been developed to extract additional information from medical images, ranging from hardware to deep learning–based image analysis solutions. This has led to the invention of radiomics, which uses data characterization algorithms to compute image features, called ‘radiomic features’ [7, 8]. These quantitative image features can accurately predict the subtype and genotypes of breast cancer [9, 10]. Furthermore, as cancer treatment becomes more individualized, DCE characteristics combined with other imaging parameters may provide more extensive prognostic information [11].

Most DCE-MRI studies have focused on measuring the intra-tumoural and background parenchymal features in individual two-dimensional (2D) slices with manually drawn ROIs [12–14]. More recently, it has been suggested that features extracted from peri-tumoural tissues could offer additional markers by reflecting angiogenic activity [15]. However, there is as yet no consensus on the optimal method of segmenting the peri-tumoural volume.

In this single-institution observational study, a semi-automatic method was developed to segment the intra-tumoural and peri-tumoural volumes of breast cancers three-dimensionally in order to analyze their pharmacokinetic properties. Our main objectives were to assess the feasibility of three-dimensional (3D) segmentation in a consecutive clinical population and to investigate whether semi-quantitative DCE parameters are associated with traditional prognostic factors. We also hypothesized that both intra-tumoural and peri-tumoural DCE parameters can be used to differentiate the breast cancers that are more aggressive from those that are less aggressive.

## Materials and Methods

### Study Design and Patients

This study was based on a database of 262 consecutive breast cancer patients prospectively included in a translational breast cancer study in 2011–2014 at our tertiary university hospital. Of these patients, the current study included those women who met the following criteria: [1] newly diagnosed invasive breast cancer; [2] pre-operative bilateral 3.0 T breast MRI; [3] mass lesions clearly demarcated on DCE-MRI; [4] healthy contralateral breast; [5] no previous history of cancer or breast operations; and [6] minimal tumour diameter of > 7 mm. This minimal diameter was selected as the threshold for inclusion to avoid possible partial volume effects in smaller structures. At our institution, breast MRI is performed according to the guidelines of the European Society of Breast Cancer Specialists working group [16]. Fifty-six patients fulfilled the inclusion criteria and are the study cohort (Table 1). Written informed consent was obtained from all the patients before any procedure. The study was approved by the Research Ethics Board of our tertiary hospital and patients provided written informed consent. All clinical investigations were conducted according to the relevant guidelines and the principles expressed in the Declaration of Helsinki.

**Table 1:** Patient profiles and tumour characteristics

Characteristic	*N* (%)
**Patients/lesions**	56/56
**Age (years)**	56.6 ± 11.0
**Menopause status**	
Premenopause	19 (33.9)
Postmenopause	37 (66.1)
**Tumour stage**	
pT1	34 (60.7)
pT2	21 (37.5)
pT3	1 (1.8)
pT4	0 (0)
**Axillary node classification**	
pN0	33 (58.9)
pN1	16 (28.6)
pN2	5 (8.9)
pN3	2 (3.6)
**Histological grade**	
G1	12 (21.4)
G2	27 (48.4)
G3	17 (30.4)
**Human epidermal growth factor receptor 2**	
Positive	45 (80.4)
Negative	11 (19.6)
**Oestrogen receptor**	
Positive	48 (85.7)
Negative	8 (14.3)
**Progesterone receptor**	
Positive	44 (78.6)
Negative	12 (21.4)
**Ki67 expression**	
< 20%	26 (46.4)
≥ 20%	30 (53.6)
**Tumour type**	
Ductal (no special type)	45 (80.4)
Lobular	8 (14.3)
Others	3 (5.4)

### Breast MRI

MRI examinations were performed in the prone position with a seven-element phased-array coil dedicated to breast imaging (Philips Achieva 3.0-T TX, Philips N.V., Eindhoven, Netherlands). The clinical structural breast MRI protocol consisted of T2-weighted and non-contrast- and contrast-enhanced 3D T1-weighted sequences and diffusion-weighted imaging. Dynamic contrast-enhanced fat-saturated 3D T1-weighted sequences (TR = 4.70 ms; TE = 2.30 ms; flip angle 10°; in-plane pixel size 0.96 × 0.96 mm; 180 slices; slice thickness 1 mm; scanning time 58.5 s) were used in this study, enhanced with an injection (0.1 mL/kg, 3 mL/s) of gadoterate meglumine (376.9 mg/mL), followed by a saline chaser. An initial pre-contrast and six (total) post-contrast sequences were used for segmentation and the subsequent assessment of the pharmacokinetic parameters.

### Assessment of Pharmacokinetic Parameters

A single observer (AN) with 4 years of experience in breast MRI analysis performed all DCE analyses, in consultation with a breast radiologist (MS) with 25 years of experience in breast radiology. For this study, the open-source image-processing package Fiji (http://fiji.sc/Fiji; in the public domain, [version 1.52p]) was used for the segmentation and analysis of the intra-tumoural and peri-tumoural pharmacokinetic properties [17]. A script was written to automate every step, except for the initial cropping of the tumour and the colour-changing procedure discussed below. Automating the process allowed a dataset of 1260 images to be analyzed in 1 min, excluding the time required to import the images from the image database because this is heavily dependent on the system used. Every patient’s 3D DCE-MRI image stack was analyzed separately. The computer used for the analysis had an Intel Core i3-6100 CPU and 16 GB of RAM. The code used in this study does not use graphics card acceleration.

### Image Analysis

#### k-Means Segmentation

A *k*-means segmentation technique [18] was used to label all voxels separately at every time point after the whole DCE-MRI image stack was deinterleaved. This method involves an unsupervised algorithm that assigns a membership to each voxel. Voxels are assigned to a cluster based on their proximity to the cluster centroids. Essentially different tissue types and backgrounds are defined based on their locations and intensity values. The clustering plug-in is based on a validated *k*-means algorithm [19].

#### Parameter Acquisition

**Fig. 1:**
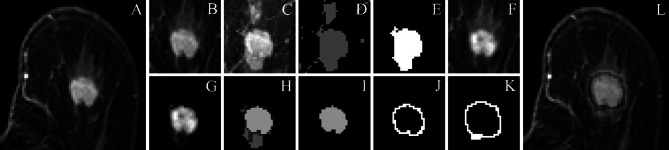
Illustration of the method. **A** Original DCE-MRI stack. **B** Cropped tumour from the last time point of post-contrast imaging. **C** Maximum intensity projection (MIP) of cropped stack. **D** *k*-means-clustered MIP. **E** Small objects removed. **F** First time point of post-contrast image stack. **G** First post-contrast image stack after application of the edited MIP mask. **H** *k*-means-clustered volume under the MIP-mask. **I** Tumour volume. **J** Peri-tumoural volume of “shell 1”. **K** Three-dimensional dilated peri-tumoural volume of “shell 2”. **L** Tumour and peri-tumoural shell masks applied to the original DCE-MRI stack

Because the method presented has multiple steps, a script was written to increase its effectiveness and to avoid tedious manual effort. The main pipeline is presented in Fig. 1. First, the image stack was imported into Fiji and an ROI was selected manually. The ROI selection was based on placing a rectangle over the tumour area in the MR slice with the largest tumour area and then stretched laterally and horizontally to ensure complete selection of the tumour. The whole DCE image stack was then deinterleaved into stacks that represented individual time points; in this case, seven stacks were made. The last post-contrast image stack was used to create a 2D maximum intensity projection (MIP), which was then segmented with *k*-means clustering. Initial clustering was carried out with two clusters with a cluster center tolerance of 0.0001, a random seed, and the image stack was interpreted as 3D. The clustered MIP was used as a mask to approximate the tumour borders coarsely by removing all the non-enhanced structures from the original 3D image stack.

After the MIP mask was applied, the *k*-means clustering algorithm was used again with four clusters to further define the edges of the tumour. The image stacks were clustered as 3D volumes into four clusters. The clusters not including tumoural tissue were removed with a colour-changing operation. Because the results of *k*-means clustering were unpredictable, this step required observer intervention. After these steps, all extra-tumoural structures were removed with a plug-in, which removed all but the largest object in the 3D stack [20]. Finally, a precise tumour volume was left. To measure the peri-tumoural volume, four stepwise-dilated tumour volumes were created and made hollow so that each of the peri-tumoural shells was one voxel thick. The shells were identified from the tumour side outwards as shells 1, 2, 3, and 4.

The intensities of the healthy background fibroglandular tissues were measured by cropping the healthy breast tissue and deinterleaving the DCE-MRI image stack. The pre-contrast stack was *k*-means clustered and the colour values of the resulting clusters were modified so that only the fibroglandular tissue was left. Because the skin and fibroglandular tissues have similar intensities, they were assigned to the same cluster when *k*-means clustering was applied. However, the skin volume was removed from the selection by eroding the clustered volume. The erosion tool works by eroding the selected volume from the outside. Because the fibroglandular tissue is in the inner part of the breast, a fixed amount of erosion could be used to remove the skin without removing FGT in any significant amount. For adipose tissue measurements, a similar *k*-means clustering method was used to extract the intensity data. These volumes of the different tissues were then used as masks to measure the intensities in the unmodified DCE-MRI stacks using a 3D intensity measurement tool [21].

### Mathematical Models

DCE-MRI data are typically interpreted based on the relationship between the intensity change and the contrast agent concentration. Although the concentration can be calculated using arterial input function methods, for this clinical study, a reference tissue method was used [22, 23].

#### Reference Tissue Method

The MRI signal intensity (*S*(*t*)) at time *t* was compared with the initial T1-weighted image intensity (*S*(0)) to obtain the contrast agent concentration (*C*(*t*)) in the selected tissue. The contrast agent concentration was quantified with Eq. 1 [24]. An approximate value was calculated by taking measurements from a reference tissue and combining the data with baseline *T*_1_ relaxation time values taken from the literature.

1 $${C}_{t}\left(t\right)\approx \frac{1}{{r}_{1}} \times \frac{1}{{T}_{1}{\left(0\right)}_{\mathrm{reference tissue}}{S\left(0\right)}_{\mathrm{reference tissue}}} \times (S\left(t\right)-S\left(0\right))$$

In our study, the adipose tissue of breasts was used as the reference tissue and a value of 366 ms (taken from the literature) was used for the adipose tissue baseline *T*_1_ relaxation time [25]. The relaxivity coefficient (*r*_1_) of the contrast agent was assumed to be constant (3.43 mM^−1^ s^−1^ at 3 T) [26]. Differences in the tissue concentrations of the contrast agent were measured with area under the curve (AUC) values and absolute concentrations (mmol/L).

#### Quantitative Kinetic Parameters

In addition to the tissue concentrations over time, the quantitative kinetic parameters were also extracted from the TICs. The initial percentage enhancement (*E*_1_), peak percentage enhancement (*E*_peak_), and signal enhancement ratio (*SER*) were calculated as follows [27]:

2 $${E}_{1}=100\% \times \frac{({S}_{1}-{S}_{0})}{{S}_{0}}$$

3 $${E}_{\mathrm{peak}}=100\% \times \frac{({S}_{\mathrm{peak}}- {S}_{0})}{{S}_{0}}$$

4 $$SER= \frac{({S}_{1}-{S}_{0})}{({S}_{\mathrm{last}}-{S}_{0})}$$

where *S*_1_ is the signal intensity in the volume of interest at the first contrast-enhanced point, *S*_peak_ is the peak signal intensity, *S*_0_ is the unenhanced signal intensity in the volume of interest, and *S*_last_ is the signal intensity in the volume of interest at the last contrast enhancement point.

### Statistical Analysis

All statistical analyses were performed with IBM SPSS Statistics for Windows, version 22 (IBM Corp., Armonk, NY, USA). Continuous variables are presented as mean ± standard deviation (SD) and categorical variables as absolute values and percentages. Two-tailed Student’s *t*-test was used to assess the differences between tumours of different grades. Pearson’s correlation coefficient was used to test the associations between continuous variables. The reproducibility of the method was tested with Cronbach’s α and intra-class correlation coefficients. Statistical significance was set at *P* < 0.05 for all tests. Given the exploratory nature of this study, we did not use the Bonferroni correction for multiple comparisons.

## Results

A total of 56 patients (mean age, 56.6 ± 11.0 years) with 56 invasive breast cancers were included in the study. The patient profiles and tumour characteristics are described in Table 1. Histopathologically, 12 patients had a low-grade (grade 1) tumour and 44 patients had a high-grade (grades 2–3) tumour.

In the intra-tumoural area, the AUC value for the concentration of contrast agent was significantly higher in the high-grade tumours (mean 27.5 mmol/L × min) than in the low-grade tumours (mean 18.3 mmol/L × min; *P* = 0.011). The mean difference was 9.14 mmol/L × min (95% confidence interval [CI] 16.1–2.20]). In the peri-tumoural area measured from the shell located closest to the tumour border (shell 1), the AUC value for the concentration of contrast agent was also significantly higher in the high-grade tumours (mean 12.3 mmol/L × min) than in the low-grade tumours (mean 8.4 mmol/L × min; *P* = 0.021). The mean difference was 3.82 mmol/L × min (95% CI 7.05–0.61).

**Fig. 2:**
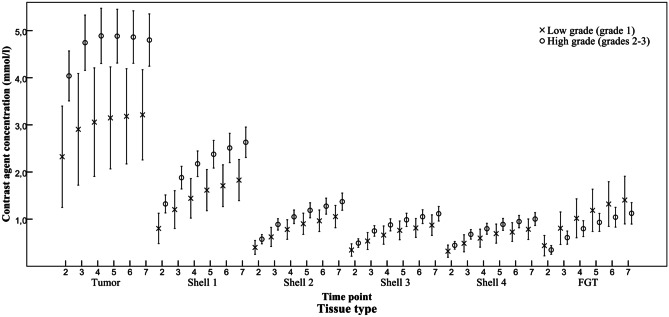
Mean contrast agent concentrations (mmol/L) in breast cancers of grade 1 vs grades 2–3. Clustered error bars at all time points. Time point 1 is in the pre-contrast phase and was not included in the measurements

The absolute concentrations of contrast material in both the intra-tumoural and peri-tumoural tissues were significantly higher in the high-grade tumours than in the low-grade tumours (Table 2 and Fig. 2). The high-grade tumours showed increased tissue concentrations of contrast agent at every time point examined. At the first post-contrast time point, the intra-tumoural concentration was 68.0% higher in the high-grade tumours than in the low-grade tumours. The absolute differences in the peri-tumoural concentrations gradually diminished in the shells situated further from the tumour border, with values of 65.0%, 53.8%, 50.0%, and 48.4% in shells 1, 2, 3, and 4, respectively.

**Table 2:** Mean contrast agent concentrations (mmol/L) of grade 1 (*n* = 12) and grades 2–3 (*n* = 44) tumours at different time points and in different regions of interest

Region	Time point	Grade 1		Grades 2 and 3		*P*
		Mean	SD	Mean	SD	
**Intra-tumoural**	2	2.47	1.61	4.15	1.86	.006
	3	3.05	1.75	4.84	1.99	.006
	4	3.40	2.01	4.97	1.96	.017
	5	3.51	1.98	4.95	1.89	.024
	6	3.46	1.73	4.92	1.83	.016
	7	3.54	1.76	4.86	1.81	.029
**Peri-tumoural shell 1**	2	0.83	0.47	1.37	0.68	.013
	3	1.28	0.63	1.93	0.85	.016
	4	1.54	0.68	2.23	0.94	.021
	5	1.73	0.74	2.43	1.00	.029
	6	1.84	0.78	2.56	1.06	.032
	7	1.97	0.80	2.68	1.09	.040
**Peri-tumoural shell 2**	2	0.39	0.21	0.60	0.34	.056
	3	0.65	0.31	0.92	0.43	.053
	4	0.79	0.30	1.08	0.51	.068
	5	0.92	0.33	1.22	0.56	.078
	6	1.02	0.37	1.31	0.60	.108
	7	1.09	0.36	1.41	0.63	.106
**Peri-tumoural shell 3**	2	0.34	0.19	0.51	0.32	.074
	3	0.54	0.26	0.78	0.40	.054
	4	0.66	0.27	0.91	0.46	.083
	5	0.77	0.28	1.02	0.50	.102
	6	0.83	0.29	1.08	0.52	.105
	7	0.89	0.31	1.15	0.54	.116
**Peri-tumoural shell 4**	2	0.31	0.19	0.46	0.29	.089
	3	0.49	0.26	0.70	0.37	.059
	4	0.60	0.27	0.85	0.42	.082
	5	0.70	0.29	0.92	0.45	.112
	6	0.73	0.28	0.87	0.47	.092
	7	0.80	0.31	1.03	0.49	.115

*SD* standard deviation

**Fig. 3:**
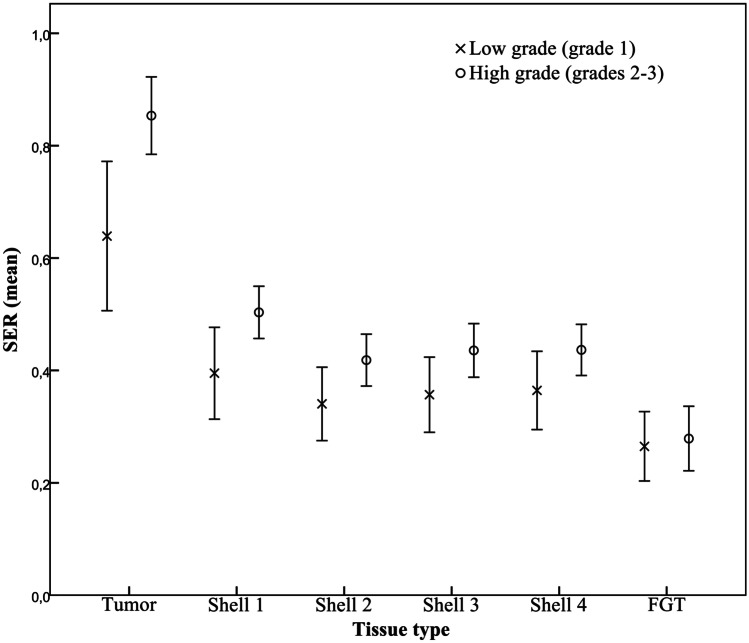
Mean *SER* values in breast cancers of grade 1 vs grades 2–3. Clustered error bars from each tissue type measured in the first post-contrast phase

The parameters derived from the TICs correlated with the histopathological parameters (Table 3 and Fig. 3). Larger tumour size correlated with higher *SER* for the intra-tumoural (*r* = 0.348, *P* = 0.009) and peri-tumoural volumes (*r* = 0.280, *P* = 0.037). Higher tumour grade correlated with higher *SER* in the intra-tumoural (*r* = 0.470, *P* < 0.001) and peri-tumoural volumes (*r* = 0.343–0.356, *P* < 0.01). Ki67 activity correlated with the intra-tumoural (*r* = 0.414, *P* = 0.002) and peri-tumoural *SER* values (*r* = 0.344–0.385, *P* < 0.05). In addition, Ki67 activity correlated weakly with the initial percentage enhancement measured in the peri-tumoural region (*r* = 0.277–0.287, *P* < 0.05). Positive oestrogen receptor status of the tumours correlated negatively with the *SER* values in the fibroglandular tissue of the healthy breast (*r* =  −0.440, *P* = 0.001). Neither the peak percentage enhancement nor the adipose tissue measurements correlated with the clinical or histopathological markers.

**Table 3:** Associations between histopathological markers and mean signal enhancement ratio (*SER*) measured in different regions

	*N*	*SER* tumour	*P*	*SER* shell 1	*P*	*SER* shell 2	*P*	*SER* shell 3	*P*	*SER* shell 4	*P*
**Tumour size**											
Large (≥ T2)	22	0.91 ± 0.23	.009	0.53 ± 0.14	.037	0.45 ± 0.18	.056	0.46 ± 0.18	.071	0.47 ± 0.16	.054
Small (≤ T1c)	34	0.74 ± 0.22		0.45 ± 0.16		0.37 ± 0.12		0.39 ± 0.12		0.39 ± 0.12	
**Histological grade**											
High [2, 3]	44	0.85 ± 0.23	.005	0.50 ± 0.15	.029	0.42 ± 0.15	.101	0.44 ± 0.16	.108	0.44 ± 0.15	.126
Low [1]	12	0.64 ± 0.21		0.40 ± 0.13		0.34 ± 0.10		0.36 ± 0.11		0.36 ± 0.11	
**ER**											
Positive	48	0.80 ± 0.25	.347	0.47 ± 0.16	.533	0.39 ± 0.15	.299	0.41 ± 0.16	.214	0.41 ± 0.15	.247
Negative	8	0.88 ± 0.15		0.51 ± 0.07		0.46 ± 0.07		0.48 ± 0.09		0.48 ± 0.10	
**PR**											
Positive	44	0.79 ± 0.23	.400	0.48 ± 0.16	.965	0.41 ± 0.16	.834	0.41 ± 0.16	.510	0.41 ± 0.15	.496
Negative	12	0.86 ± 0.29		0.48 ± 0.13		0.40 ± 0.11		0.44 ± 0.12		0.45 ± 0.13	
**HER2**											
Positive	45	0.87 ± 0.25	.322	0.51 ± 0.21	.539	0.42 ± 0.14	.705	0.44 ± 0.14	.565	0.44 ± 0.15	.677
Negative	11	0.79 ± 0.24		0.47 ± 0.14		0.40 ± 0.15		0.41 ± 0.15		0.42 ± 0.14	
**Ki67**											
High (≥ 20%)	30	0.90 ± 0.22	.002	0.53 ± 0.16	.003	0.45 ± 0.15	.009	0.47 ± 0.16	.005	0.47 ± 0.15	.004
Low (< 20%)	26	0.70 ± 0.22		0.42 ± 0.13		0.35 ± 0.12		0.36 ± 0.12		0.36 ± 0.11	
**LNM**											
Positive	23	0.88 ± 0.23	.073	0.53 ± 0.16	.049	0.44 ± 0.19	.143	0.46 ± 0.19	.087	0.46 ± 0.18	.109
Negative	33	0.76 ± 0.23		0.45 ± 0.14		0.38 ± 0.10		0.39 ± 0.10		0.40 ± 0.11

Higher intra-tumoural contrast agent concentrations (> 3.5 mmol/L at time point 2; > 4.0 mmol/L at time point 3) achieved a positive predictive value (PPV) of over 90% for the detection of high-grade tumours. A similar PPV was also observed using the contrast agent concentrations in the peri-tumoural shell 1 (Table 4).

**Table 4:** Performance of the intra- and peri-tumoural tissue contrast agent concentration as a marker to classify tumours as low- or high-grade tumours

Tissue (time point)	Sensitivity (%)	Specificity (%)	Accuracy (%)	PPV (%)	NPV (%)	Threshold (mmol/L)
Tumour, 2	65.9	75	67.9	90.6	37.5	3.5
Tumour, 3	68.2	75	69.6	90.9	39.1	4.0
Shell 1, 2	65.9	75	67.9	90.6	37.5	1.2
Shell 1, 3	56.8	83.3	62.5	92.6	34.5	1.8

*PPV* positive predictive value, *NPV* negative predictive value, *Threshold* the tissue contrast agent concentration used to divide tumours into low- and high-grade tumours

The intra-observer reproducibility of the method was high. For the intra-tumoural intensity measures, a mean Cronbach’s *α* value of 0.995 was achieved between all time points. For the peri-tumoural intensity measures, mean Cronbach’s *α* values of 0.974, 0.995, 0.998, and 0.998 were achieved for shell 1, shell 2, shell 3, and shell 4, respectively. The Cronbach *α* values for the intra- and peri-tumoural contrast agent concentrations (0.969–0.993) were similar to those for the intensity measures. The Cronbach *α* values for the adipose and fibroglandular tissue intensities were 0.905 and 0.999, respectively. The intra-class correlation coefficients for intra-rater consistency are shown in Table 5.

**Table 5:** Intra-observer reproducibility of measurements assessed with intra-class correlation coefficients. Single measures and two-tailed analysis of consistency

	ICC	Lower	Upper	Sig
Tumour	.986	.949	.996	< .001
Shell 1	.968	.886	.991	< .001
Shell 2	.990	.965	.997	< .001
Shell 3	.994	.978	.998	< .001
Shell 4	.996	.984	.999	< .001
Adipose	.826	.477	.950	< .001
FGT	.998	.994	1.000	< .001

## Discussion

DCE-MRI is widely used in clinical breast cancer imaging. The tumour enhancement patterns have been studied extensively and many mathematical models have been developed to represent the dynamic flow and accumulation of contrast agent. However, there are still few studies of the enhancement patterns in the peri-tumoural region. In this study, we developed a method to measure the 3D intensity data in both the intra- and peri-tumoural regions, quantified as absolute concentrations. The kinetic parameters were also extracted from the time-signal curves. Our results show that not only did the low- and high-grade tumours differ in their intra-tumoural enhancement characteristics, but that the differences in the peri-tumoural region were also significant. Both the intra-tumoural and peri-tumoural contrast agent concentrations proved to have a high positive predictive value in the classification of tumours with a higher histopathological grade. These findings suggest that peri-tumoural pharmacokinetic parameters can be used as additional surrogate markers in future multi-parametric statistical analyses to model and predict tumour aggressiveness and prognoses, and to measure their responses to neoadjuvant treatments.

We analyzed several parameters (time intensity curves (TIC), the initial percentage enhancement (*E*_1_), peak percentage enhancement (*E*_peak_), and signal enhancement ratio (*SER*)) that represent the dynamic flow and accumulation of contrast agent in breast tumours and found that high tumoural and peri-tumoural contrast agent concentrations and high *SER* values are associated with tumour aggressiveness. High-grade tumours showed markedly higher tissue concentrations of contrast agent than low-grade tumours at every time point. The *SER* values of the intra-tumoural and peri-tumoural volumes correlated with tumour grade, size, and Ki67 activity, which are all markers for poor prognosis. Both the *SER* value and the absolute contrast agent concentration in the tumoural and peri-tumoural tissues may be useful as imaging indices in the characterization of tumour aggressiveness.

Currently, there is no consensus on the optimal imaging biomarkers with which to quantify the aggressiveness or prognostic characteristics of breast cancers. Earlier studies showed that even the most basic qualitative parameters (e.g. the shape of the TIC) correlate significantly with the more sophisticated quantitative perfusion parameters (e.g. *K*_Trans_, *K*_ep_, and *V*_e_) [28], More research on the predictive value of the existing imaging parameters, with the inclusion of new markers, is required to establish an optimal multi-parametric MRI prognostication tool.

The peri-tumoural region has recently received increasing attention in the field of cancer imaging. The peri-tumoural area supports the wound response-like process, the inflammatory response, the increased vascular density, and the permeability of the vessels of the tumour, and consists of an extracellular matrix and various cell types [29]. Peri-tumoural markers are associated with the response to neoadjuvant chemotherapy [12, 30, 31], hyaluronan accumulation, and lymph node metastasis [13, 14, 32, 33], pathological biomarkers [34], prognosis [35, 36], and the molecular subtype of the tumour [37]. However, the methods used to measure and extract data from the peri-tumoural region vary widely.

Manual segmentation has been used in many ways of even the most recent publications. Several studies have used a hand-drawn ROI placed on a single 2D slice for manual tumour delineation [12–14], whereas other studies have included multiple slices [30, 31] or even the whole tumour [33–35]. However, manual segmentation is time-consuming and highly operator-dependent, and thus leads to great variability between operators. Therefore, an automatic and user-independent method is required to generate more reproducible, reliable, and comparable results and to save time. Automated segmentation has been used in several studies to minimize operator error [37, 38]. The present method uses standardized 3D measurements with minimal user input, which minimizes intra- and inter-observer measurement bias, and results in excellent internal consistency. The method is also computationally undemanding, and can even be run on low-end computers.

The study limitations included the low number of patients analyzed, and the lack of access to a T1-mapping or arterial input function. However, variability is reported to be low when reference tissue models are used [22, 23, 39]. Despite these limitations, we detected significant differences between low- and high-grade tumours, highlighting the relevance of quantifying the pharmacokinetic parameters of breast tumours. Specifically, assessing the quantitative parameters in the peri-tumoural region as an adjunct to intra-tumoural assessments is a useful component of the DCE analysis.

In conclusion, characterizing breast cancer and its biomarkers as precisely as possible is important in the era of personalized medicine because treatment modalities are tailored to individuals. Our results demonstrate that the 3D segmentation model is feasible and offers an easy and standardized method of evaluating dynamic pharmacokinetic parameters, not only for tumoural areas but also for peri-tumoural areas. Low- and high-grade tumours differ significantly in their intra-tumoural and peri-tumoural enhancement characteristics. These findings should encourage the use of pharmacokinetic parameters as surrogate markers in future statistical analyses to model and predict the aggressiveness and prognosis of tumours, and to measure their responses to neoadjuvant treatments.
